# Retraction Note: Type IV collagen α1-chain noncollagenous domain blocks MMP-2 activation both in-vitro and in-vivo

**DOI:** 10.1038/s41598-020-76500-9

**Published:** 2020-11-26

**Authors:** Yakkanti Akul Sudhakar, Raj Kumar Verma, Smita C. Pawar

**Affiliations:** 1 Cell Signaling Laboratory, Bioscience Division, Center for Cancer and Metabolism, SRI International, Menlo Park, CA 94025 USA; 2 Irma Lerma Rangel College of Pharmacy, Texas A&M Health Science Center, Kingsville, TX 78363 USA; 3 Department of Genetics, Osmania University, Hyderabad, AP 500007 India; 4 Cell Signaling and Tumor Angiogenesis Laboratory, Department of Genetics, Boys Town National Research Hospital, Omaha, NE 68131 USA

Retraction of: *Scientific Reports* 10.1038/srep04136, published online 26 March 2014

The Editors have retracted this article.

An investigation by the US Office of Research Integrity (ORI) [1] has concluded that the protein band arresten (α1(IV)NC1) in Figure 3C (lanes 3–6) was reused in the grant applications R01 CA143128-04 and R01 EY024967-01 to represent a different set of experiments. The ORI was unable to determine which, if any, of these data are correct. The authors have not been able to provide any of the requested original data for this study. The Editors no longer have confidence in the integrity of the data presented in this article.

Yakkanti Akul Sudhakar & Smita C. Pawar disagree with the retraction. The Editors were not able to obtain a current email address for Raj Kumar Verma.
